# Bladder exstrophy in adulthood: About a case report

**DOI:** 10.1016/j.eucr.2022.102001

**Published:** 2022-01-13

**Authors:** Seif Mokadem, Ahmed Saadi, Bilel Saideni, Mohamed Ben Salah, Abderrazak Bouzouita, Mohamed Chebil

**Affiliations:** a University of Tunis El Manar, Faculty of Medicine, Charles Nicolle Hospital of Tunis, Urology Department, Tunisia; b University of Tunis El Manar, Faculty of Medicine, Charles Nicolle Hospital of Tunis, Orthopedics Department, Tunisia

**Keywords:** Bladder exstrophy, Adulthood, Malformation, Abdominal wall

## Abstract

Bladder exstrophy is a severe malformation characterized by the lack of the anterior sub-umbilical abdominal wall, and the front wall of the bladder. We present a rare case of a 26-year-old woman without any previous medical or surgical history, that we treated for bladder exstrophy. We performed an iliac osteotomy, bladder enlargement using the ileum and a Monti-type continent urinary derivation and a Promentofixation. A vesico-cutaneous fistula was diagnosed after surgery and we failed to manage it after two surgical revision. Therefore, we performed a cystectomy and a non-continent Bricker external urinary derivation.

## 1 Introduction

Bladder exstrophy is a severe malformation characterized by the lack of the anterior sub-umbilical abdominal wall, and the front wall of the bladder, as well as affecting the urethra, pelvic floor, the external genital organs, and the perineum.^1^ While efforts are focused on the antenatal assessment of the bladder exstrophy, some patients have never been diagnosed with this condition despite its impact on their lives. The syndrome has particular features in adulthood: it becomes a psychological, social, and professional disability.^1^ There are also surgical challenges making this malformative pathology special in adults, such as the complexity of parietal fixation and frequent need for osteotomy, as well as the importance of preserving the upper urinary tract.^1^

## 2 Case presentation

**Fig. 1:**
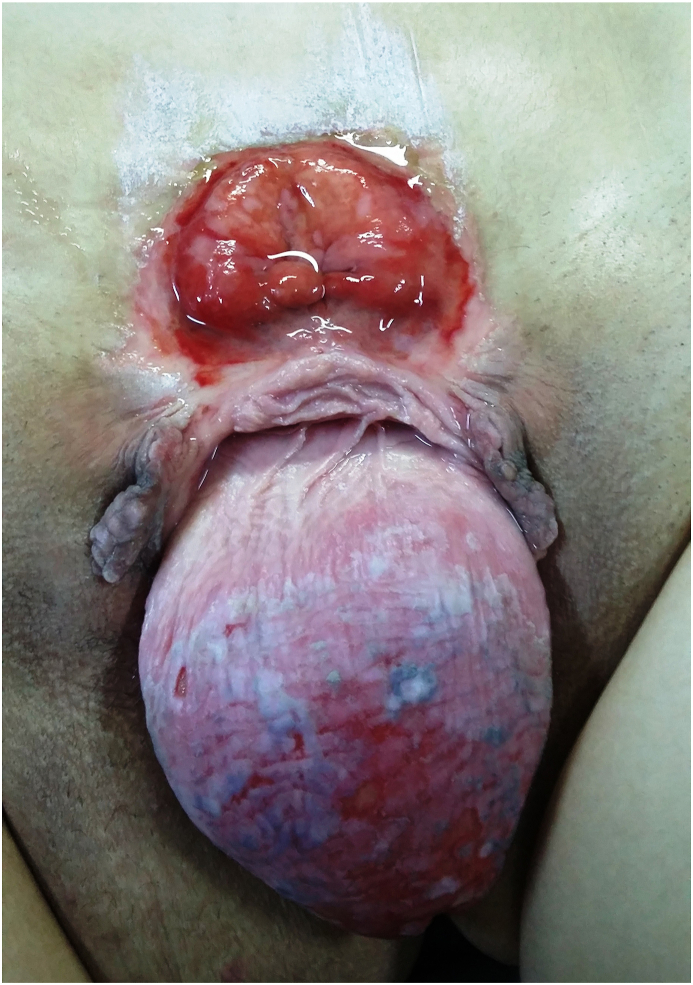
First examination.

We present the case of a 26-year-old woman without any previous medical or surgical history who presented adulthood (just before marriage) for never-treated bladder exstrophy. On examination, we discovered an inflammatory hypogastric bladder plate measuring 7 cm (Fig. 1). We also found two clitoral halves and a fourth-grade uterine prolapse (Fig. 1). A pubic diastasis was also observed (Fig. 1).

**Fig. 2:**
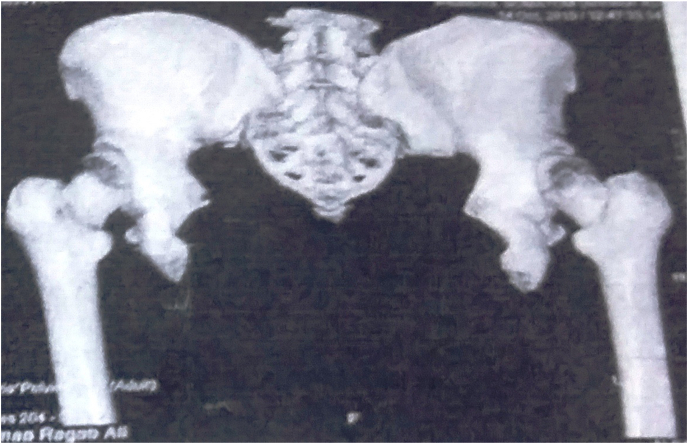
X-ray of the pelvis.

**Fig. 3:**
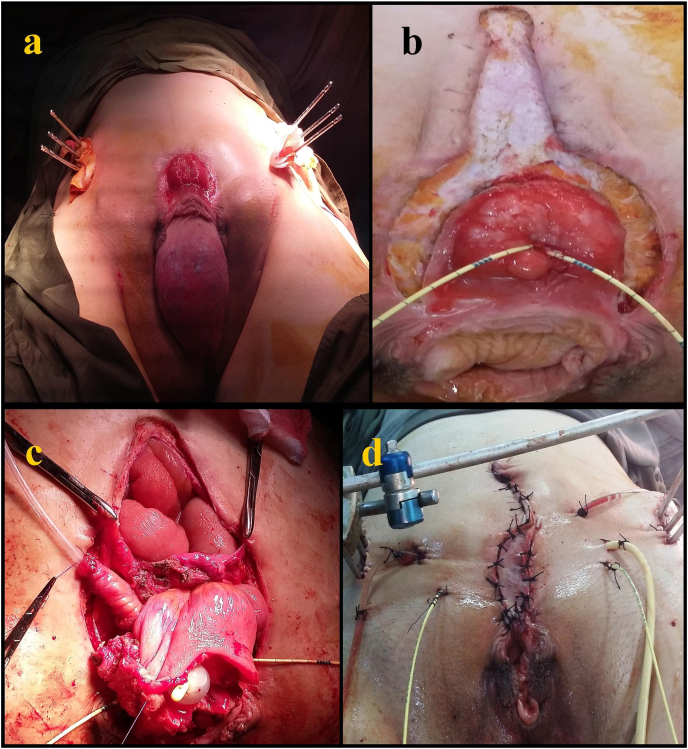
a:Iliac osteotomy and an implant of external fixation on both iliac bones, b: Liberation of the bladder plate, c: Bladder enlargement using the ileum and a Monti-type continent urinary derivation, d: Immediate result after surgery.

On X-ray of the pelvis, we found the typical abnormalities of bladder exstrophy, including lateral rotation and shortening of the pubic bone, and a pubic diastasis (Fig. 2). On CT scan the two kidneys had normal morphology. We decided to perform surgery in cooperation with the orthopedic surgery team. The surgical management was as follows:

The first step consisted of an iliac osteotomy and an implant of external fixation on both iliac bones (Fig. 3a). We then placed two ureteral stents and liberated the entire bladder plate, preserving the anterior and inferior pedicles (Fig. 3b). Then we performed a Promentofixation of the vaginal fundus and the uterine isthmus. Next, we proceeded to a bladder enlargement using the ileum and a Monti-type continent urinary derivation (Fig. 3c). We performed a wall reconstruction for the closure using a vascular skin flap. The drainage consisted on two ureteral catheters, a cystostomy catheter, a Monti probe, and a Retzius drain (Fig. 3d). Following the surgery, we experienced necrosis of the skin flap at Day14, which evolved well under directed healing. The Retzius drain was extracted at D6 and the ureteral catheters at Day28 and Day29. Monti catheter was removed and self-clean catheterization was started at Day32. A vesico-cutaneous fistula was diagnosed at Day38 and the patient underwent a revision surgery at Day45 to excise a fistula communicating between the skin and the anterior bladder wall and perform double drainage using a cystostomy tube and the Monti system. Second revision surgery was performed at Day59 due to the persistency of the fistula. She underwent double ureteral tube drainage, double nephrostomy, and closing of the fistula. However, the patient kept a high flow recurrent vesico-cutaneous fistula. Therefore, We performed a cystectomy and a non-continent Bricker external urinary derivation and abdominal wall reconstruction with Vicryl plate after 5 months. There were no post-operative outcomes. We noticed the recurrence of uterine prolapse at 14 months follow-up. The long-term follow-up was marked by the recurrence of the prolapse at 14 months post-op making any spontaneous pregnancy impossible.

## 3 Discussion

Exstrophy of the bladder is a complete defect of the urogenital sinus and the skeletal system. The hypogastric area is occupied by the inner surface of the posterior wall of the bladder, whose mucosa is fused with the skin.^1^

Total reconstruction of bladder exstrophy in neonatal period is treatment of choice with good results. Expert centers with high recruitment volume may have better results. Extrophy in adulthood is very rare, as the obvious leaking urine cannot be unnoticed.

Management of bladder extrophy in adults is a surgical challenge. Good knowledge of different methods of anterior abdominal wall reconstruction following cystectomy or cystoplasty is mandatory. Urinary diversion and abdominal wall closure in one surgical procedure without osteotomy is feasible and potentially successful.^1^ Few cases of bladder extrophy in adults have been reported.

Matsuda et al.^2^ reported a case of a bladder exstrophy in an adult female, treated with cystectomy, construction of a continent ileal reservoir, and closure of the abdominal fascial defect using alloplastic material. This surgery improved her quality of her life.

Gulati et al.^3^ reported the cases of two adult females presenting with untreated bladder exstrophy underwent cystectomy and modified Mainz pouch. Both these patients had improved quality of life and renal function. Ozdiler et al.^4^ reported a 49-year-old female who presented with bladder extrophy. Bilateral uretero-sigmoidostomy was performed and the patient was discharged ten days later in a good condition. Evaluation of upper urinary tract 6 months following operation showed a marked improvement in its dilatation. Quattara et al.^5^ also reported a 39-year-old male who had presented with bladder extrophy. Due to the lack of evidence, there are no guidelines that help us to choose between continent or incontinent urinary diversion.

The surgical management of bladder extrophy in adults is complex and postoperative complications are possible as in our patient. The aim of this management must be to obtain continence and, at least, preserve the nephrological prognosis.

## 4 Conclusion

Adult bladder extrophy is a real sexual, psychological and social tragedy for patients. The improvement of the prognosis depends on early treatment, if possible from birth, aiming at reconstructing a urinary and genital system as close to normal as possible.

## Consent

Signed consent was obtained from the patient.

## Declaration of competing interest

The authors declare that there are no conflicts of interest regarding the publication of this article.
